# Nanobubble-embedded inorganic 808 nm excited upconversion nanocomposites for tumor multiple imaging and treatment†
†Electronic supplementary information (ESI) available: The characteristics of NB, UCNP and UCNP–CN@NB show the basic state of the nanomaterials. The further energy level diagrams, lifetime and electric field distribution show the energy transfer from UCNP to CN. The cell *in vitro* analysis is also disclosed. See DOI: 10.1039/c8sc00108a


**DOI:** 10.1039/c8sc00108a

**Published:** 2018-02-09

**Authors:** Ming-Hsien Chan, Yu-Ting Pan, Yung-Chieh Chan, Michael Hsiao, Chung-Hsuan Chen, Lingdong Sun, Ru-Shi Liu

**Affiliations:** a Department of Chemistry , National Taiwan University , Taipei 106 , Taiwan . Email: rsliu@ntu.edu.tw; b Genomics Research Center , Academia Sinica , Taipei 115 , Taiwan . Email: mhsiao@gate.sinica.edu.tw; c Department of Biochemistry , College of Medicine , Kaohsiung Medical University , Kaohsiung , 807 Taiwan; d Beijing National Laboratory for Molecular Sciences , State Key Laboratory of Rare Earth Materials Chemistry and Applications , PKU-HKU Joint Laboratory in Rare Earth Materials and Bioinorganic Chemistry , College of Chemistry and Molecular Engineering , Peking University , Beijing 100871 , China; e Department of Mechanical Engineering and Graduate Institute of Manufacturing Technology , National Taipei University of Technology , Taipei , 106 Taiwan

## Abstract

Contrast reagents for ultrasound imaging are widely used in clinical medical diagnosis because ultrasound resolution is limited. Contrast agents must be utilized to enhance the image resolution. At present, microbubbles (MBs) and nanobubbles (NBs) are the main contrast reagent candidates for improving the signal resolution. Fluorescence upconversion nanoparticles provide high sensitivity and also function as nanocarriers. This can label tumor cells in a specific organ under irradiation of near-infrared (NIR) light. However, despite the use of NIR light, the penetration depth of NIR is only approximately 15 mm. Thus, we combine fluorescence with ultrasonic imaging to achieve the effect of multiple imaging and solve the low penetration depth of fluorescence imaging and the poor resolution of ultrasound imaging. The dual imaging modalities achieved higher resolution or signal to noise ratios. In this study, Nd^3+^-sensitized upconversion nanoparticles (UCNPs) are combined with graphitic carbon nitride quantum dots (CNs) and embedded in NBs (UCNP–CN@NBs). The UCNPs are excited by 808 nm light and emit visible and ultraviolet light. Then, the energy of the ultraviolet light is transferred to the CNs to produce reactive oxygen species (ROS) for photodynamic therapy. Ultrasonic waves are also used to promote NB bursting and the release of ROS molecules in photodynamic therapy, leading to cancer cell apoptosis.

## Abstract 2

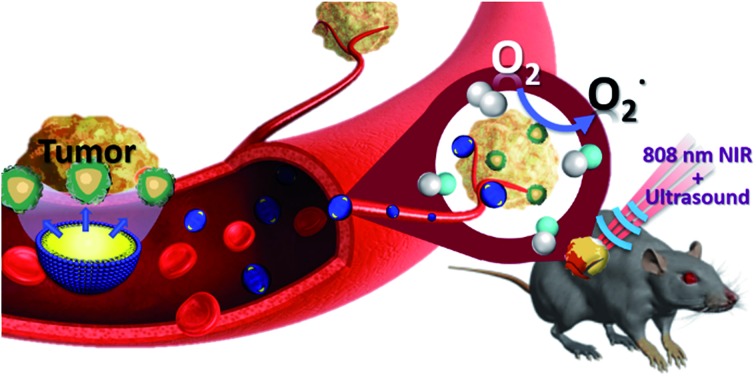
Upconversion nanocomposites embedded in nanobubbles can be a new technique to achieve imaging and therapy under 808 nm irradiation.

## Introduction

By the mid-twentieth century, ultrasound was widely used in the medical field because it can penetrate the muscles and soft tissues. Ultrasound scanning does not involve harmful radiation and is generally used for scanning organs to help in medical diagnosis and treatment.^1^ However, acoustic impedance is similar in various body organs, thereby resulting in poor resolution of the ultrasound image.^2^ Ideal ultrasound contrast agents exhibit high biocompatibility, can be readily metabolized, and possess a significantly different acoustic impedance in biological tissues. Based on the above characteristics, microbubbles (MBs) and nanobubbles (NBs) are considered suitable as ultrasound contrast agents.^3^ Adding MBs enhanced the contrast of ultrasound images and helped detect the amount and flow rate of blood using Doppler imaging to assess the tumor condition.^4^ However, cavities within the tumor tissues are approximately 400–600 nm.^5^ Compared with MBs, NBs are considered to be a better candidate because of the size limitation of ultrasound contrast agents.^6^,^7^ Despite the use of contrast agents image resolution remains insufficient to replace other detection systems. Every detection method has advantages and disadvantages. Thus, the development of multiple imaging can combine two or more imaging modes at the same time to minimize the respective disadvantages of each method.^8^–^11^ Compared with ultrasound imaging, fluorescence imaging technology has a very high sensitivity. However, it has the disadvantage of insufficient penetration depth. Traditional progress in fluorescence imaging has been primarily based on down-converted materials which emit visible light when they absorb short wavelength and high frequency-excited light sources such as ultraviolet light. Ultraviolet light causes damage to biological tissues and its use is limited because of low penetration.^12^,^13^ In contrast to that previously described, upconversion nanoparticles (UCNPs) are capable of absorbing long-wavelength light and emitting short-wavelength light by energy conversion.^14^ The excitation light source of these materials is mainly near-infrared (NIR) light. NIR light avoids damage to biological tissues and enables the penetration depth to become deeper than that when using ultraviolet and visible light. NIR light also possesses the advantages of high-spatial resolution, a narrow emission band and low biotoxicity. It is widely used in biomedical applications such as in fluorescent probes, biological imaging, disease diagnosis and treatment.^15^ Studies also reveal the use of UCNPs as a light source carrier to produce ultraviolet and visible light by NIR light excitation. Through this fluorescence-resonance energy transfer, UCNPs can transfer energy to the photosensitizer at a specific wavelength and achieve successful photodynamic therapy (PDT).^16^,^17^ Inorganic photosensitizers, such as titanium dioxide (TiO_2_) and graphitic carbon nitride quantum dots (CNs), display extraordinary catalytic activity and biological stability because of their appropriate band gap and high specific surface area. Most notably, the electronic structure of CNs can provide a visible light response upon ultraviolet irradiation, which demonstrated the potential of CNs as a photosensitizer for the generation of reactive oxygen species (ROS) for PDT.^18^ As a cancer treatment, PDT is non-invasive and can be used in combination with traditional chemotherapy and radiation therapy.^19^,^20^ PDT can generate reused ROS and kill cancer cells specifically without damaging the surrounding normal tissues. PDT combines optoelectronic technology and biomedical treatment.^21^ This kind of therapy must gather three basic elements, namely, a light source, a photosensitizer and oxygen. Although PDT shows some excellent therapeutic advantages, the issues of (1) a lack of light penetration depth,^22^ (2) poor stability of organic photosensitizers,^23^ and (3) the hypoxia that commonly surrounds cancer cells still need to be discussed in future research.^24^ In this study, Nd^3+^-sensitized UCNPs were combined with graphitic phase carbon nitride (CNs) dots and embedded in oxygen-containing NBs (UCNP–CN@NBs). When UCNP–CN@NBs are irradiated with 808 nm NIR light, the UCNPs transfer the excited energy to the CNs which produce ROS by absorbing the upconverted ultraviolet light.^25^ Ultrasound is then used to break the bubbles. The released ROS can damage the cancer cells, allowing the cancer cells entry to the apoptosis pathways.

**Fig. 1:**
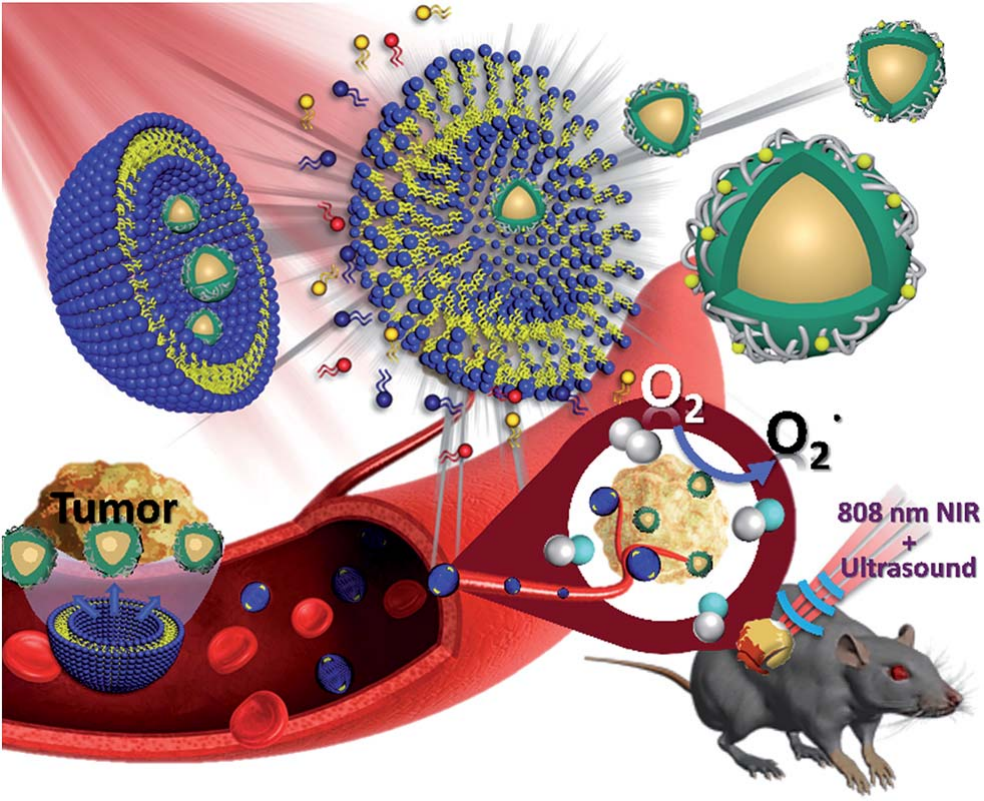
Schematic illustration of the UCNP–CN@NBs applied in PDT upon laser irradiation.

This study improves the three basic elements of PDT as follows: (1) for light source, the use of 808 nm light within the biological NIR window prevents absorption by hemoglobin and water molecules and results in a good light penetration depth.^22^ (2) In the choice of photosensitizer, inorganic CNs are characterized by high biocompatibility and photoluminescence (PL) properties. (3) Mixing oxygen in the NBs increases the ROS production and solves the state of hypoxia around the cancer cells. After a series of *in vitro* and *in vivo* studies the use of the UCNP–CN@NB nanoplatform proved to be a successful and intelligent design to attain good results *via* the combination of multiple imaging and light therapy (Fig. 1).

## Results and discussion

### Characterization of UCNP–CN@NBs

**Fig. 2:**
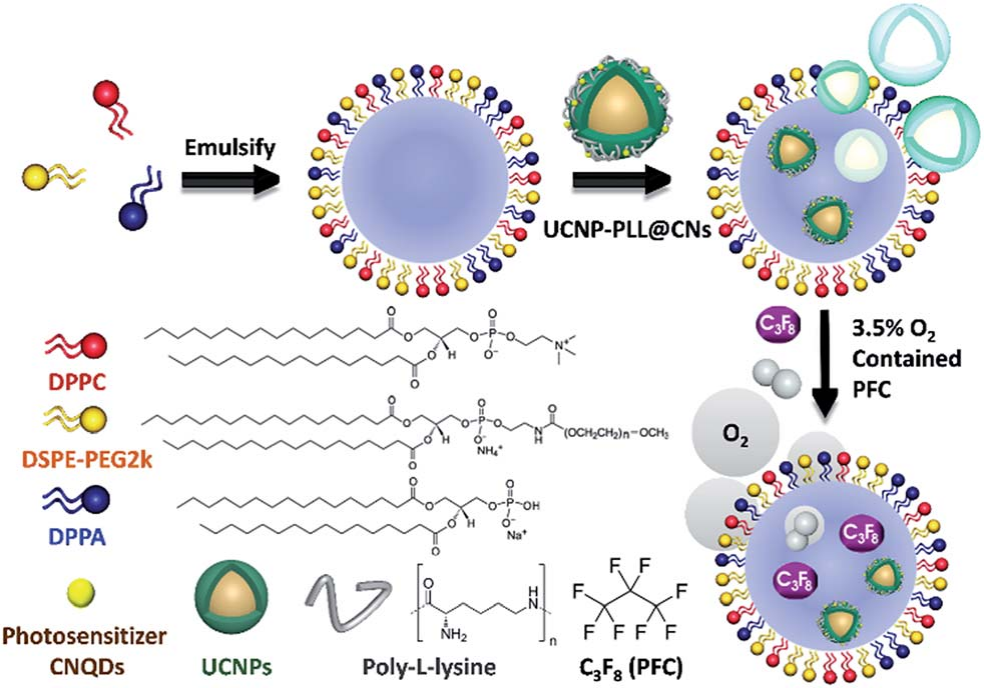
Experimental steps of the synthesis of upconversion nanocomposites embedded in nanobubbles (UCNP–CN@NBs).

In this study, we used a micelle synthetic method to uniformly mix DPPC, DSPE-PEG2000 and DPPA.^26^ The micelle precursor was synthesized through the homogeneous process, followed by the addition of octafluoropropane gas (C_3_F_8_) to form stable NBs as shown in Fig. S1a.† The embedding of the upconverted nanocomposites in the NBs is similar to the previous steps. The only difference is that when the interfacial active agent dispersed uniformly the upconverted nanocomposites were added to the mixture. We also adjusted the ratio of C_3_F_8_ and oxygen. The upconverted nanocomposites were embedded in oxygen-containing NBs, named UCNP–CN@NBs (Fig. 2). We chose to add 3.5% oxygen into the bubbles to ensure the bubbles are still stable in water. If the embedding ratio of oxygen is higher than 5%, the bubbles will be unstable and broken. First, we carried out a series of NB and upconverted nanocomposite material identification tests. To evaluate NB morphology by scanning electron microscopy (SEM), 10 μL of the NBs was dropped onto a glass sheet. The aqueous solution was evaporated to form a thin film at room temperature. Then, because the surface of the NBs is not a conductive metal, we increased the NB conductivity by sputtering with platinum (Pt) to cover the lipid shell and analysed the NBs by SEM.^27^ In Fig. S1b† the NB structure reveals a uniform spherical shape and the average size is approximately 285 nm.

Moreover, the NBs are broken in the vacuum environment after Pt sputtering (Fig. S1c†). As well as SEM investigation, transmission electron microscopy (TEM) and laser scanning confocal microscopy (LSCM) were also used to analyse the lipid layer structure on the NB surface. The lipid layer structure can be seen by TEM imaging (Fig. S1d†). To confirm whether the lipid structure is stable, DiI dye was doped into the lipid layer.^28^ Under LSCM, DiI shows a red fluorescence at a wavelength of 580 nm under 561 nm laser excitation and marks the NB location. In Fig. S1e and f,† we can observe the lipid layer structure of the NBs, marked by circular red fluorescence in the dark and bright field. Co-precipitation, thermal decomposition and hydrothermal processes are commonly used methods for UCNP synthesis. In this study, the UCNPs were synthesized by a high-temperature co-precipitation method, which can produce spherical UCNPs with uniform particle size.^29^ The other material, graphitic phase CNs, was synthesized as a photosensitizer for PDT by a hydrothermal method. Urea and sodium citrate were used as the source of nitrogen and carbon, respectively. After dialysis and preserving in water solution, the CNs were conjugated with the UCNPs by a PLL polymer. A similar process as demonstrated in the previous study was used to generate UCNP–PLL@CNs. The morphology of the UCNPs and UCNP–PLL@CNs can be obtained using TEM and energy-dispersive X-ray spectroscopy (EDS). In Fig. S2a,† the diameter of the core of the UCNPs is shown to be 26 ± 1 nm. To synthesize the 808 nm-excited UCNPs within the core–shell structure, we added the Nd^3+^-doped shell structure.^30^ The diameter of the 808 nm-excited UCNPs is 29 ± 1 nm (Fig. S2b†). Comparing the core and core–shell UCNPs, the shell thickness is approximately 3 nm, and the nanoparticles were homogeneously dispersed in aqueous solution with no significant size difference. The UCNP–PLL@CNs were characterized by high-resolution TEM (Fig. S2c†) and EDS (Fig. S2d†). The high-angle annular dark-field scanning transmission electron microscopy-EDS mapping shows that Nd is located in the shell structure and dispersed in the periphery of the nanoparticles. Tm in the core layer gathers at the center of the nanoparticles. Meanwhile, F, Y and Yb are uniformly dispersed in the core and shell layers. The N in the CNs can be uniformly dispersed on the surface of the upconverted nanoparticles. X-ray diffraction (XRD) of the UCNPs was used to identify the X-ray crystallographic properties of the composites (Fig. S3a†). In the crystallization evaluation, the XRD patterns were compared to determine whether the hexagonal phase changed during the synthetic process (Fig. S3b†).

**Fig. 3:**
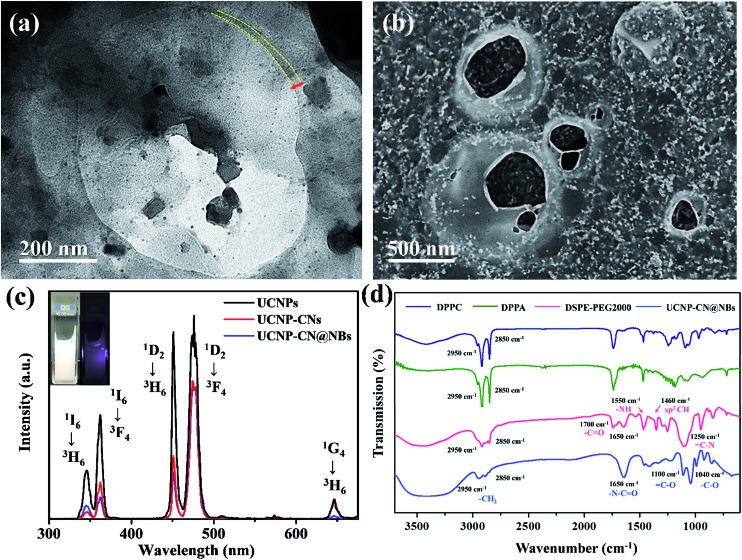
(a) TEM and (b) SEM images of UCNP–CN@NBs. (c) UV-vis and photoluminescence spectra of UCNP–CN@NBs. UCNP–CN@NBs were irradiated by a 808 nm laser at 0.5 W cm^–2^. (d) FTIR spectra of UCNP–CN@NBs.

In this study, the data of the hexagonal β-NaYF_4_ (JCPDS 16-0334) were compared with those of the UCNPs, UCNP–PLL and UCNP–PLL@CNs. The diffraction peaks of the UCNPs, UCNP–PLLs and UCNP–PLL@CNs were similar to those of the standard. This was followed by optical analysis and Fig. S3c† shows the absorption spectrum of the CNs and the emission spectra of the UCNPs. In the ultraviolet light band at 345 nm, fluorescence was observed from the UCNPs and absorption was also observed for the CNs. The overlapping in the ultraviolet region between the UCNPs and the CNs reveals the possibility of fluorescence resonance energy transfer (FRET). In this study, Fourier transform infrared (FTIR) spectroscopy was also used to detect the functional groups of UCNP–PLL@CNs. UCNP–PLL@CNs show a characteristic absorption at 1440 cm^–1^ because of the stretching vibration of the CO bond. The absorption peak at 1130 cm^–1^ was caused by the amino groups of the PLL molecule and the C–N bond of the CNs. The information for the characteristic FTIR peaks is shown in Fig. S3d.† The vibrational spectra show that the PLL molecule and the CNs were bound with UCNPs. Subsequently, the size and morphology of UCNP–CN@NBs can be detected by TEM. Given that the composition of the NBs has no conductive metal, we use 2% phosphotungstic acid to prepare the negative sample staining.^31^ The experimental results are shown in Fig. 3a. The upconverted nanocomposites were contained in NBs. In addition, the lipid layer structure of the NBs can also be observed in Fig. 3a marked with a yellow curve. As indicated previously, SEM can also be used to detect the UCNP–CN@NBs morphology. As previously illustrated in Fig. S1c,† because no conductive metal exists on the NB surface, we also sputtered UCNP–CN@NBs with Pt as shown in Fig. 3b. The broken bubbles contained UCNPs inside. Furthermore, UCNPs scattered around the bubbles. We speculate that the vacuum situation might cause the bubbles to burst and release the packaged UCNPs, resulting in the UCNPs being distributed inside and outside the bubbles.

### Evidence of ultrasound and fluorescence imaging

**Fig. 4:**
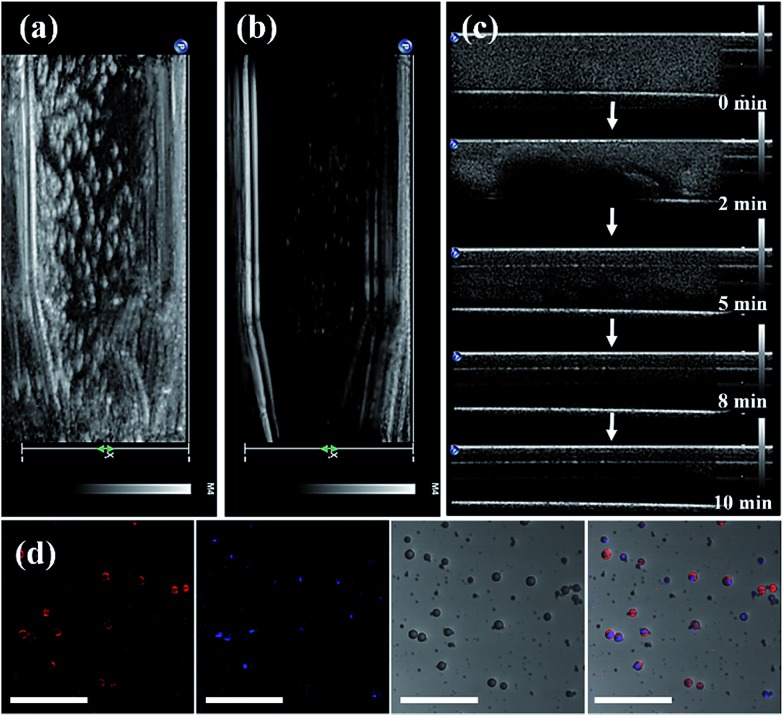
Experimental setup for detecting the burst time of the bubbles. The bubbles are present in (a and b) the Eppendorf and (c) the glass tube. (d) Laser scanning confocal microscopy images of UCNP–CN@NBs (red fluorescence: DiI, blue fluorescence: UCNPs, scale bar: 10 μm).

To understand the optical properties of UCNP–CN@NBs, Fig. 3c illustrates the real fluorescence spectra of the UCNPs, UCNP–CNs and UCNP–CN@NBs. The inset picture shows that UCNP–CN@NBs are a white turbid solution under the white lamp. When UCNP–CN@NBs are irradiated at 808 nm using a NIR laser, a blue-violet emission can be observed. We selected the CNs, which are matched with the characteristic ultraviolet emitting peaks of UCNP–CN@NBs, as the photodynamic photosensitizers. Fig. 3c shows that the ultraviolet 345 and 365 nm bands of the UCNPs significantly decline because of the CN absorption.^32^ In addition, the PL spectrum also demonstrates that when the nanocomposites are embedded in the NBs, the other emission peaks of the UCNPs slightly decrease because of the NB shielding. The lipids contain several unique functional groups. Fig. 3d shows the FTIR spectra of DPPC, DPPA, DSPE-PEG2000 and UCNP–CN@NBs. The wavenumbers of 2850 and 2950 cm^–1^ display the characteristic absorption peaks, which are the expanded C–H bond of the aldehyde group (O=CH_2_). The UCNP–CN@NB composition was then matched with that of the three lipid precursors. The 1650 cm^–1^ characteristic absorption is NC=O, thereby indicating that the unique functional group of DSPE-PEG2000 can be detected. The 1100 and 1040 cm^–1^ absorptions are =CO and –CO vibrations caused by band stretching. The three lipids that compose the NBs all contain this functional group, confirming that the UCNP–CN@NBs are composed of these three lipid molecules. We also confirmed the blasting time of UCNP–CN@NBs at room temperature and under infrared light. In Fig. S4a and b,† because of the protective effect of the C_3_F_8_ gas, the bubbles can be retained for about two weeks without being ruptured. In addition, irradiation by the 808 nm laser for 20 min does not cause any damage to the bubbles. Dynamic light scattering (DLS) and zeta potentials can be used to identify particle size and surface electrical properties, respectively. The DLS measurement results are shown in Fig. S4c.† The average hydration radius of UCNP–CN@NBs is 428 nm. The size of the bubbles is uniform and fixed between 400 and 600 nm. The zeta potential measurement is shown in Fig. S4d.† Furthermore, we also use DLS to focus on the breaking of UCNP–CN@NBs (Fig. S5a†). The nanobubbles and UCNP nanocomposites were kept for 21 days in PBS and medium (Fig. S5b and c†). The results match with those of the previous test that the bubbles can be retained for about two weeks and not be ruptured. The average interface potential of UCNP–CN@NBs is –84 mV. For ultrasound imaging, the use of UCNP–CN@NBs as the contrast agent to enhance the resolution was investigated using an ultrasonic scanner (Philips iU22). The ultrasound imaging is shown in Fig. 4a and b. We placed gel between the probe and the Eppendorf tube to avoid the influence of air.

In Fig. 4a, we use the ultrasonic frequency of 7 MHz to produce the white dot-like ultrasonic signal, which is homogeneously distributed in the Eppendorf tube. Our result indicates that the NBs can enhance the ultrasonic signal because of the different sound impedance compared to the liquid. After 10 min of treatment, the signal of bubbles vanished (Fig. 4b). Moreover, we also injected UCNP–CN@NBs into the water to detect the burst rate of the bubbles. The probe is immersed in water containing the sample, and the signal is detected at different times. In Fig. 4c, the white signal of the bubbles is mainly distributed in the aqueous solution, and the signals can also be found at 2 min. With increasing time the white signal is reduced in 5 min and disappears after 10 min. After 10 min of treatment the bubbles faded. From this series of tests we have proven the burst rate of UCNP–CN@NBs. Thus from now on, we will treat with ultrasound for 10 min to break the bubble and release the nanocomposite. We then also used LSCM to detect UCNP–CN@NBs and to try to understand the state of the composites in a biological simulation environment. The DiI dye marked the bubble location and the UCNPs were excited with a 808 nm laser and blue light at a wavelength of 478 nm was detected. In contrast to the TEM image in Fig. 1, it can be estimated that there are about 8–10 UCNP nanocomposites in every bubble with a size of about 300 nm. As shown in Fig. 4d, the red fluorescence of DiI surrounded the outer periphery of the bubbles.

The blue fluorescence of the UCNPs was located at the center of the bubbles. The results show that the UCNP composite is embedded in the NBs. In order to further confirm that the inorganic composites were embedded in the nanobubbles, we added the upconversion composites before micelle formation. However, since the structure of the lipid layer has been built, the upconversion composites cannot enter into the bubbles even if the shielding gas (C_3_F_8_) is added (Fig. S6†).

### Evaluation of ROS effect *in vitro*

**Fig. 5:**
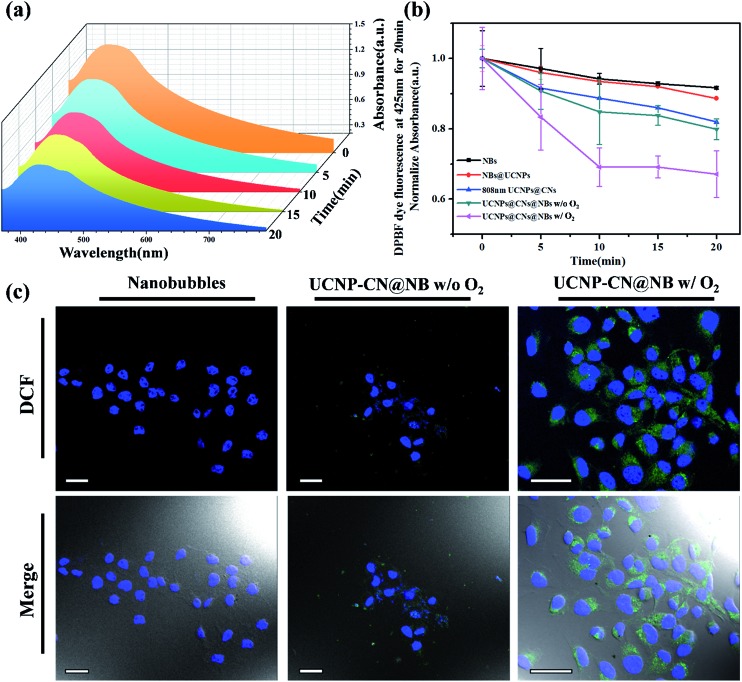
Laser excitation of UCNP–CN@NBs at 0.5 W cm^–2^ for 30 min can produce reactive oxygen species (ROS). (a) UCNP–CN@NBs containing O_2_ can decrease the absorption of the ROS probe, DPBF, with increasing time. (b) The linear trend of the UV/vis absorption spectrum of DPBF. (c) Cells treated with UCNP–CN@NBs (500 μg mL^–1^) were irradiated by an 808 nm laser and stained with DCF. An increase of green fluorescence in irradiated cells suggests ROS production (scale bar: 25 μm).

In PDT light irradiation causes the photosensitizer to produce ROS and thus treatment is achieved. The ROS effect can be quantitative and/or qualitative as chemical reagents act with ROS *via* a series of chemical reactions because ROS are highly active. In this study, the DPBF reagent was used as a ROS indicator to detect ROS in UCNP–CN@NBs in the solution. The absorption coefficient of DPBF can be obtained using a UV-visible light absorption spectrometer. During the process, 20 μL of the DPBF reagent was added to 1 mg mL^–1^ UCNP–CN@NB liquid. The mixture was placed in a cuvette and irradiated with NIR light and continuously stirred. The NIR light irradiation was used after ultrasound treatment for 10 min to break the bubbles. The UV/visible light absorption spectrometer was then used to detect the change in the absorption peak of DPBF. In Fig. 5a we chose NBs, NBs with O_2_, UCNP–PLL@CNs, UCNP–CN@NBs and UCNP–CN@NBs with O_2_ under irradiation using an 808 nm laser for 0, 5, 10, 15 and 20 min. When more ROS existed in the liquid, the absorption of DPBF greatly decreased due to the changes in the structure of the DPBF reagent.^33^ When 808 nm laser irradiation was used for 20 min, its absorption value decreased by 45%. In this study, the ROS production of a series of samples was also measured by the change of the DPBF reagent at 425 nm. As a result, the longer the irradiation time, the more ROS were produced by the sample (Fig. 5b). After irradiation for 20 min no significant decrease in absorption was observed for the NBs and UCNP@NBs. However, the ROS production of UCNP–CN@NBs with O_2_ and without O_2_ shows different results. UCNP–CN@NBs with O_2_ show the highest ROS production. Meanwhile, the absorption value of DPBF decreased to almost 40%. Based on the above, although UCNP–CNs, UCNP–CN@NBs and UCNP–CN@NBs with O_2_ exhibit the ability to produce ROS, UCNP–CN@NBs with O_2_ can generate the most ROS because of the O_2_ content. Before their application in the biological field, it is necessary to evaluate the biocompatibility of all these materials and investigate whether these materials are toxic or not. Therefore, we chose 3T3 (mouse fibroblast cell line) and Huvec (human umbilical vein endothelial cells) cell lines as normal cell lines to evaluate the toxicity of the NBs and UCNP–CN@NBs. Moreover, OECM-1 (oral cancer cells) and CAL-27 (tongue squamous cell carcinoma cells) cell lines were also selected as the controlled cancer cell lines. These two oral cancer cell lines have different properties and the OECM-1 cells were used as *in vitro* models due to being sensitive to PDT. However, they cause the production of large amounts of tissue fluid during tumor transplantation in mice. Thus, we chose CAL-27 cells as the *in vivo* model. The results are shown in Fig. S7a–d.† Despite increasing the sample concentration to 250 μg mL^–1^, the viabilities of all cells remained at approximately 80%. To understand the relationship between UCNP–CN@NBs and the cells and to confirm whether these nanocomposites can be internalized by the cells, the endocytosis effect of the cells was observed by LSCM (Fig. S8†). We administered 100 μg mL^–1^ UCNPs, NBs and UCNP–CN@NBs and co-cultured them with OCEM-1 cells.

**Fig. 6:**
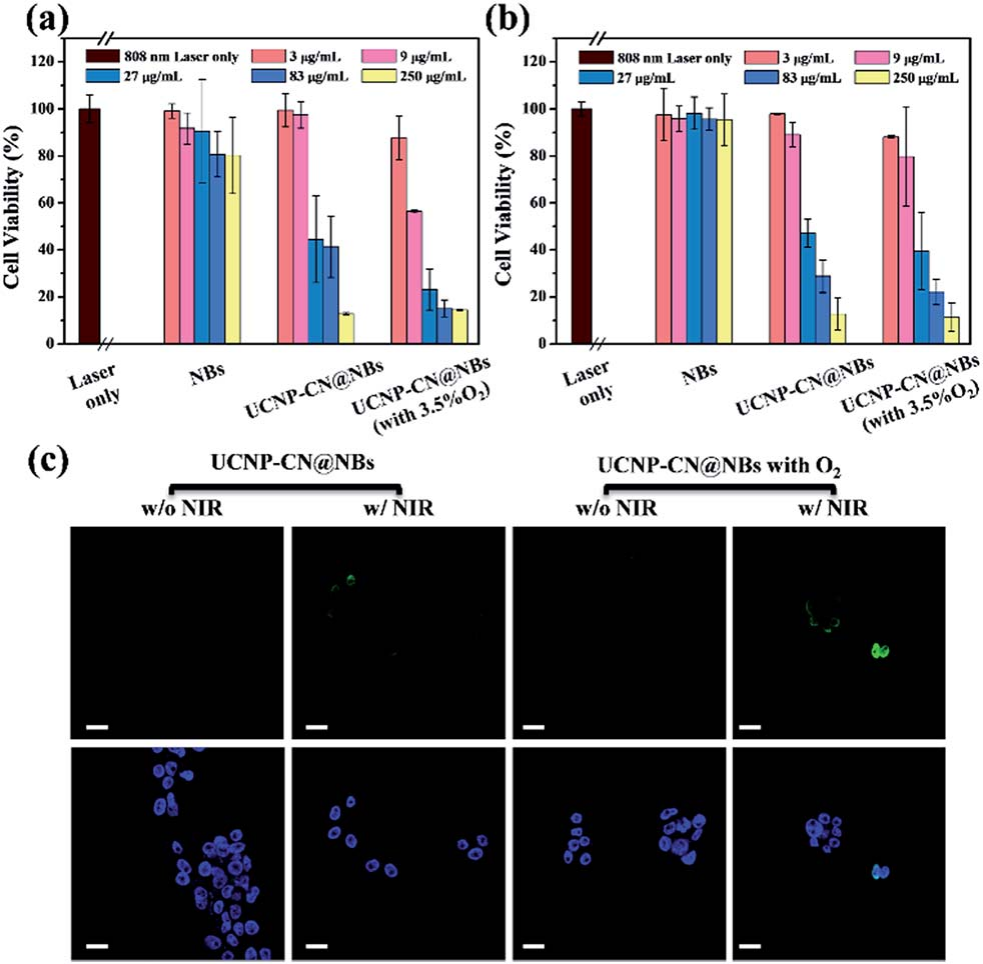
Cytotoxicity after treatment with NBs, UCNP–CN@NBs and UCNP–CN@NBs containing O_2_ for (a) the OECM-1 cell line and (b) the CAL-27 cell line after 808 nm laser irradiation for 5 min. (c) TUNEL assay of OECM-1 cells treated immediately with NBs, UCNP–CN@NBs and UCNP–CN@NBs containing O_2_ under 808 nm laser irradiation for 10 min (scale bar: 25 μm).

We first doped NBs with DiI so that the lipid layer can be observed under LSCM. As a result, DAPI uniformly stained the nuclei. The blue fluorescence shows the portion of OECM-1 cancer cells. To avoid the difficulty of distinguishing the blue light from the UCNPs, the upconverted emission light was displayed using green fluorescence. The three kinds of materials are located around the nucleus or overlap with the cytoplasm. This test shows that the UCNP–CN@NB nanocomposites can be ingested into the cancer cells through the endocytosis pathway. After analyzing ROS in the solution, confirming whether ROS can be effectively produced after the NIR light irradiation to achieve the PDT effect is necessary. In this study, the 2′,7′-dichlorofluorescin diacetate (H_2_-DCFDA) reagent was used to detect ROS production in cells. The H_2_-DCFDA reagent is not a fluorescent dye and is free to pass through the cell membrane. When the H_2_-DCFDA reagent enters into the cell cytosol, it can be hydrolyzed to 2′,7′-dichlorofluorescin (DCFH) by the lipolytic enzyme named intracellular esterase. DCFH cannot penetrate the cell membrane. Thus, DCFH remains in the cell cytosol. When ROS exist in the cell environment, DCFH will be oxidized into a fluorescent dye, DCF. The detection principle is based on DCF detection, which can emit green fluorescence allowing us to determine the ROS content in cells.^34^ In Fig. 5c, the green fluorescence intensity indicates the ROS levels. We excited DCF using a 498 nm laser and it can emit 530 nm visible light. In this study, OECM-1 cells were selected and the LSCM images show the ROS content of the NBs and UCNP–CN@NBs with O_2_ and without O_2_ after 808 nm laser (0.5 W cm^–2^) irradiation for 10 min. After laser irradiation for 10 min, both UCNP–CN@NBs with O_2_ and without O_2_ generate a green fluorescence. Moreover, the fact that UCNP–CN@NBs with O_2_ produce more ROS than UCNP–CN@NBs without O_2_ can be distinguished by comparing the merge results with the bright-field. These results indicate that UCNP–CN@NBs can withstand the hypoxic environment of the OECM-1 cells and produce more ROS than in previous research. In addition to observing the ROS production of cells by LSCM, we also performed a cytotoxicity test to confirm the PDT activity. The concentrations of NBs, UCNP–CN@NBs with O_2_ and UCNP–CN@NBs without O_2_ were set to 3, 9, 27, 83 and 250 μg mL^–1^. The experimental procedure used 808 nm laser irradiation (0.5 W cm^–2^). The cytotoxicity test results are shown for OECM-1 cells (Fig. 6a) and CAL-27 cells (Fig. 6b). The survival rate of cancer cells with UCNP–CN@NBs with O_2_ and without O_2_ decreased with increasing sample concentration. UCNP–CN@NBs with O_2_ has a higher half maximal inhibitory concentration (IC_50_) than that of UCNP–CN@NBs without O_2_. We then examined the apoptosis of the OECM-1 cells induced by UCNP–CN@NBs with O_2_ and without O_2_ using the TUNEL method (Fig. 6c), and selected fluorescein as a fluorescent marker.^35^ After 10 min of 808 nm laser irradiation, the fluorescence of fluorescein isothiocyanate (FITC) was detected by LSCM at 450 nm, showing apoptosis. The FITC fluorescence intensity shown confirms that UCNP–CN@NBs with O_2_ has a more significant signal than UCNP–CN@NBs without O_2_. The results show that UCNP–CN@NBs with O_2_ can induce cancer cell apoptosis in OECM-1 cancer cells and overcome the hypoxia of cancer cells to produce more ROS and achieve a better PDT effect.

### Evidence of multiple imaging and PDT for *in vivo* studies

**Fig. 7:**
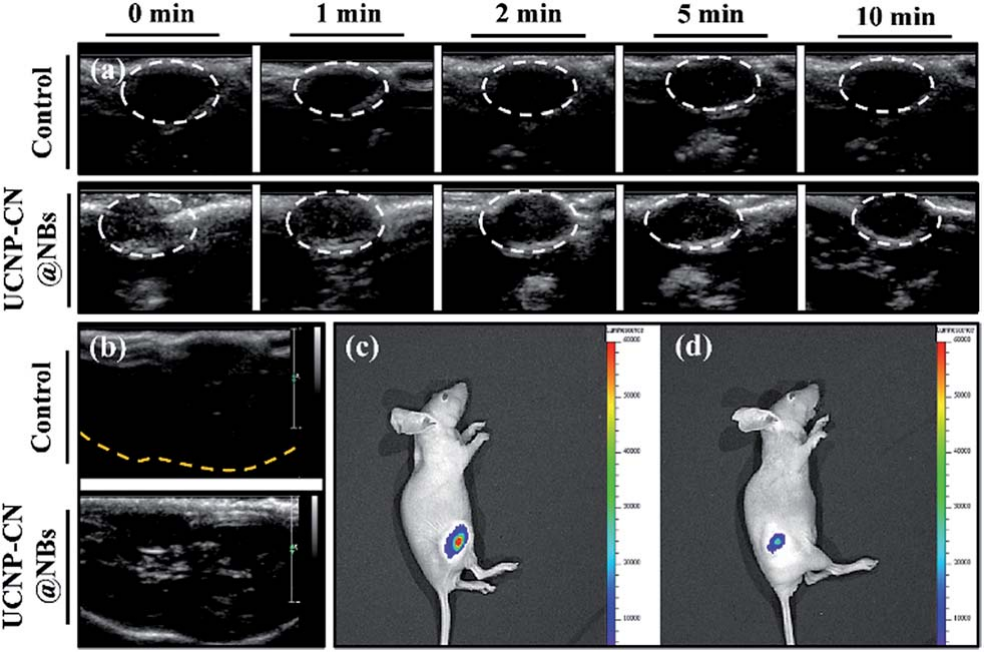
(a) Ultrasound imaging of the animal model. The test time from 0 min to 10 min was used to evaluate the time at which UCNP–CN@NBs were broken. (b) Focus ultrasound signal can be found indicating that UCNP–CN@NBs greatly increase the tumor contrast. (c and d) The fluorescence in the animal model can be detected by IVIS. The mouse on the left was injected with UCNPs only, whereas the one on the right was injected with UCNP–CN@NBs.

**Fig. 8:**
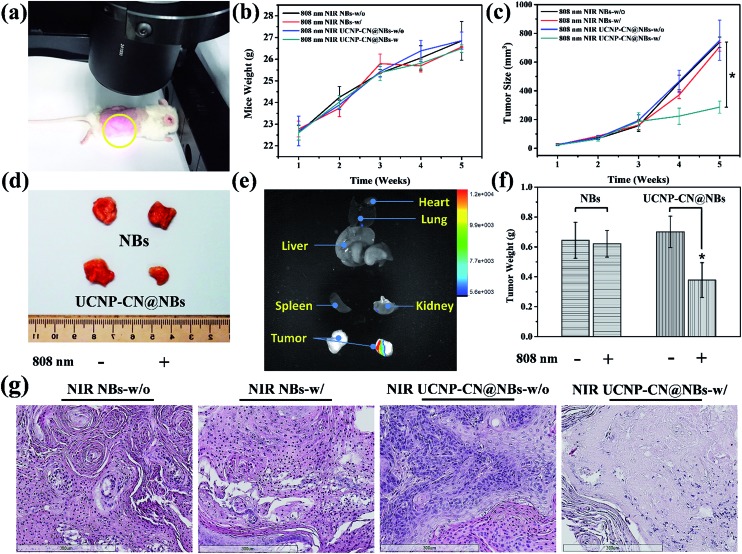
(a) Photograph of the set-up for the PDT treatment *in vivo* test. (b) Mouse weight with time for NBs and UCNP–CN@NBs with or without 808 nm treatment. (c) Tumor size with time for NBs and UCNP–CN@NBs with or without 808 nm treatment. (d) Photograph showing the tumor sizes after the different treatments. (e) The fluorescence imaging of major organs. The left tumor is injected with NBs as a control group and the right tumor is injected with UCNP–CN@NB as the experimental group. (f) Relative tumor weights after injection with NBs or UCNP–CN@NBs before and after the 808 nm laser treatment. (g) Haematoxylin and eosin (H&E) staining of the tumor tissue carried out after the NIR treatments. Symbols and error bars are mean ± S.D. for four tumors per group (*n* = 4, *p < 0.05).

We also evaluated ultrasound imaging and fluorescence imaging *in vivo*. In Fig. 7a, the ultrasound signal can easily be seen when UCNP–CN@NBs are injected in the tumor cells. Focus imaging also can obtain high contrast after UCNP–CN@NB injection (Fig. 7b). Moreover, the fluorescence signal can be identified by a non-invasion *in vivo* imaging system (IVIS). The mouse on the left side was injected with UCNP before 808 nm laser treatment (250 mW cm^–2^). The 475 nm blue fluorescence can be observed using a CCD camera in an IVIS chamber (Fig. 7c). In comparison, the mouse on the right side was injected with UCNP–CN@NBs and the fluorescence intensity slightly decreased because of the shielding effect of bubbles (Fig. 7d). In order to increase the accumulation of UCNP nanocomposites, we synthesized NBs to transport the nanomaterials into the cells. The *in vivo* PDT tests in this study used a higher concentration of samples because of the low toxicity of the lipid structure on the surface of the NBs. This concentration has both the weight of the NBs and the UCNP nanocomposite. The concentration of the UCNPs should still be within acceptable limits. The mice were classified into groups either with or without irradiation using an 808 nm laser. The real situation of the PDT *in vivo* test is shown in Fig. 8a. With the tumor size being about 125 mm^3^, sample doses (40 mg kg^–1^ each) were given for two groups: the NBs as a control group and the UCNP–CN@NBs with O_2_ as the experimental group. The individual tumors were exposed to an 808 nm laser with beam expander at 250 mW cm^–2^ for 30 min. The sample was injected once on the third week and irradiated under a NIR laser. The tumor size results are shown in Fig. 8c. Laser treatment showed that only UCNP–CN@NBs under laser irradiation could inhibit tumor growth during the fourth and fifth weeks, during which the tumors were smaller (approximately 200 mm^3^) compared with the other group. The weight of the mouse did not change when PBS and UCNP–CN@NBs were injected with or without laser irradiation after two weeks. This result indicates that the treatment did not adversely affect the health of the mouse (Fig. 8b). The mouse was sacrificed on the fifth week because the tumor overgrowth was too large. The tumor weight and size with UCNP–CN@NBs after laser treatment were reduced by almost twofold (Fig. 8d), which shows the excellent PDT efficacy. The remarkable difference in therapeutic performance between the measured tumor size in the mice before sacrifice and the real tumor size after sacrifice could be attributed to the severe inflammatory phenomenon inside the tumor. Moreover, we also used an IVIS system to detect the fluorescence of major body organs (Fig. 8e). The test conditions are the same as in the previous description. We injected both sides of the mice with different materials and excised the tumor at the fifth week. The left tumor was injected with NBs as a control group and the right tumor was injected with UCNP–CN@NB as the experimental group. No significant material accumulation in other organs was shown in the experimental group, and the tumor size of the experimental group is also smaller than that of the control group. In Fig. 8f, we show the tumor weight to further ensure the treatment result. Compared with NBs, UCNP–CN@NBs have extremely limited the growth of the tumor. Haematoxylin and eosin (H&E) staining of the tumor tissue was carried out after the NIR treatments (Fig. 8g). As expected, significant damage to cancer cells was noticed only in the UCNP–CN@NBs with 808 nm irradiation group. The other groups reveal a tumor morphology with distinctive membrane and nuclear structures.

## Experimental section

### Materials

All chemicals were used without further purification. The phospholipids used in the NB synthesis included 1,2-dipalmitoyl-*sn-glycero*-3-phosphocholine (DPPC), 1,2-dihexadecanoyl-*sn-glycero*-3-phosphate (DPPA), and 1,2-distearoyl-*sn-glycero*-3-phosphoethanolamine-*N*-[methoxy(polyethylene glycol)-2000] (DSPE-PEG2000) in powder form, which were purchased from Avanti Polar Lipids. Chloroform (CHCl_3_) was purchased from Midland Scientific. Glycerol (C_3_H_8_O_3_) and cyclohexane (C_6_H_12_) were purchased from J.T. Baker. Pluronic F-127 and rare-earth hydrate acetates, including Y(CH_3_CO_2_)_3_·H_2_O, Yb(CH_3_CO_2_)_3_·H_2_O, Tm(CH_3_CO_2_)_3_·H_2_O, and Nd(CH_3_CO_2_)_3_·H_2_O, were purchased from Aldrich. 1-Octadecene (C_18_H_36_, ODE, 90%), oleic acid (C_18_H_34_O_2_, OA, 90%), ammonium fluoride (NH_4_F), sodium hydroxide (NaOH, 98%), hydrochloric acid (HCl, 37%), ethanol (C_2_H_6_O, 99%), urea powder (NH_2_CONH_2_, 98%), poly-l-lysine (PLL) hydrobromide, 1,3-diphenylisobenzofuran (DPBF), 2′,7′-dichlorofluorescin diacetate (H_2_-DCFDA), 1,1′-dioctadecyl-3,3,3′,3′-tetramethylindocarbocyanine perchlorate (DiI), and the cell death detection kit were purchased from Sigma-Aldrich. Trisodium citrate (Na_3_C_6_H_5_O_7_, 99%) was purchased from Acros. Dulbecco’s Modification of Eagle’s Medium (DMEM) and 4′,6-diamidino-2-phenylindole (DAPI) were purchased from Invitrogen. The triple antibiotic reagent (penicillin–streptomycin–glutamine) was purchased from GIBCO. Alamar Blue cell viability reagent was purchased from Thermo Fisher Scientific.

### Nanobubble (NB) synthesis

The NBs were prepared using a thin-film hydration-sonication method. First, all phospholipids were dissolved in 4 mL CHCl_3_ and formed a thin phospholipid film by natural evaporation. The thin-film was then hydrated with a 4 mL hydration solution which contained 10% C_3_H_8_O_3_ and 2 mg mL^–1^ Pluronic F-127. This process was performed in a shaking incubator for 1 h at 37 °C to prepare the liposomes. The liposomal suspension was then added into a round-bottomed flask. Subsequently, octafluoropropane and nitrogen, which ensure that only C_3_F_8_ gas surrounds the NBs, were added to the flask. Finally, the probe of the Sonic VC 750 ultrasonic sonicator was placed at the air–liquid interface. The solution was then sonicated for 2 min to form the NBs. Samples were stored at 4 °C.

### Synthesis of NaYF_4_:Yb^3+^,Tm^3+^ core upconversion nanoparticles (UCNPs)

The NaYF_4_:Yb/Tm UCNPs were synthesized by a high-temperature coprecipitation method. Firstly, the precursor of NaYF_4_:Yb/Tm was synthesized by mixing 0.64 mmol Y(CH_3_CO_2_)_3_, 0.144 mmol Yb(CH_3_CO_2_)_3_ and 0.016 mmol Tm(CH_3_CO_2_)_3_ with 6 mL OA and 14 mL ODE. The mixture was heated to 110 °C to evaporate the water. This mixture was then heated to 150 °C for 1 h and cooled down to room temperature. The precursor was synthesized in this step. After synthesizing the precursor, 0.1475 g NH_4_F and 0.1 g NaOH were added to the precursor and were mixed well in methanol. This reaction mixture was reacted in a 100 mL round-bottomed flask, heated at 120 °C to remove the methanol, and then heated at 290 °C for 2 h. The mixture was slowly cooled to room temperature and centrifuged with C_2_H_6_O. The precipitate was stored in C_6_H_12_ at 4 °C.

### Synthesis of NaYF_4_:Yb^3+^,Tm^3+^@NaYF_4_:Nd^3+^,Yb^3+^ core–shell upconversion nanoparticles (808 nm UCNPs)

The synthesis of NaYF_4_:Yb/Tm@NaYF_4_:Yb/Nd was similar to the synthesis of NaYF_4_:Yb/Tm. NaYF_4_:Yb/Tm@NaYF_4_:Yb/Nd was the core–shell UCNPs. The different steps used 0.64 mmol Y(CH_3_CO_2_)_3_, 0.24 mmol Yb(CH_3_CO_2_)_3_ and 0.24 mmol Nd(CH_3_CO_2_)_3_ as the shell precursor. After the shell precursor was synthesized, the core UCNPs (NaYF_4_:Yb/Tm) and the methanol solution, which contained 0.1475 g NH_4_F and 0.1 g NaOH, were added. The reaction was reacted in a 100 mL round-bottomed flask similar to that of synthesized NaYF_4_:Yb/Tm, heated at 120 °C and then heated at 290 °C for 2 h. After heating for 2 h, the mixture was slowly cooled to room temperature and centrifuged with C_2_H_6_O. The precipitate was stored in C_6_H_12_ at 4 °C.

### Modification of the 808 nm UCNPs with CNs (UCNP–CNs)

To combine the 808 nm UCNPs (NaYF_4_:Yb/Tm@NaYF_4_:Yb/Nd) with graphitic carbon nitride quantum dots (CNs), we used PLL. Firstly, we used 0.05 M alcohol-diluted HCl to remove the OA and ODE ligands on the UCNP surface. After sonication, the ligands were removed and the compounds were transferred to a water phase. Secondly, we added an aqueous solution of PLL and mixed with the UCNPs for 24 h at room temperature. The solution was then centrifuged three times. The precipitates were suspended in water. Finally, 0.5 mL CNQD solution was mixed well with 0.5 mL of the previous solution for 2 h and centrifuged with water twice. After washing with water, the nanocomposites, UCNP–CNs, were suspended in 0.5 mL water.

### Synthesis of the UCNP–CN@NB nanocomposites

The process of synthesizing UCNP–CN@NBs is similar to that for NBs. When the thin phospholipid film was formed, the surfactant, which contained 10% C_3_H_8_O_3_ and 2 mg mL^–1^ Pluronic F-127, was mixed with the thin film. At the same time, we added 0.4 mL UCNP–CN into the solution. This process was performed in a shaking incubator at 37 °C for 1 h to prepare the liposomes. The liposomal suspension was then added into a round-bottomed flask. Subsequently, octafluoropropane, which contained 3.5% oxygen, and nitrogen were added to the flask. We synthesized the bubbles containing 3.5% oxygen as the bubbles would be unstable and broken if the ratio of oxygen was higher than 5%. The 3.5% oxygen can ensure the bubbles are still stable in water. The ultrasonic sonicator probe was placed at the air–liquid interface. The solution was sonicated for 2 min to form UCNP–CN@NBs. The aqueous solution was dialyzed against deionized water through a dialysis membrane (300 KD, mPES) for 12 h to remove the unembedded nanocomposites and then stored at 4 °C.

### Photodynamic effect experiment

DPBF was used to detect ROS by reacting with ^1^O_2_ in the aqueous solution. 1 mg mL^–1^ NBs, UCNP@NBs, UCNP–CNs, UCNP–CN@NBs and UCNP–CN@NBs with O_2_ were mixed well with 10 μL DPBF, placed in a cuvette and stirred. Each sample was separately irradiated with a 0.5 W cm^–2^ 808 nm laser at different times, such as 5, 10, 15 and 20 min. After being irradiated using an 808 nm laser, the sample was irradiated by ultrasound at a frequency of 7 MHz for 10 min to ensure nanobubble explosion. The ultrasonic scanner is a Philips iU22 with L15-7io as the transducer probe. Moreover, transducers need to be added to the ultrasonic glue to block air contact. ROS production was confirmed by detecting the absorption band of DPBF. DPBF reacted irreversibly with ^1^O_2_ to reduce the intensity of the absorption band at approximately 425 nm. After irradiation by the 808 nm laser and ultrasound, the absorption spectra of the sample were recorded using a UV-1700 spectrophotometer (Shimadzu, Japan).

### Cellular uptake and localization of UCNP–CN@NBs

In this study, the biological tests were performed in cell lines *in vitro* and in a mouse model *in vivo*. No human subjects were used in the entire study. All experiments were performed in compliance with the relevant laws or guidelines. For the *in vitro* model, oral adenosquamous and oral epidermoid carcinoma cell lines (the CAL-27 cell line and the OECM-1 cell line) were selected in this research. The two cell lines were seeded in six-well plates at a density of 20 000 cells per mL and incubated overnight. The cell lines were then incubated with the previous samples for 12 h at 37 °C. We use confocal laser scanning microscopy to confirm the sample localization. The cells were washed using phosphate-buffered saline (PBS) after incubation to remove the samples which were not internalized in the cells. We then added 1 mL 4% paraformaldehyde per well for 5 min to fix the cells on the glass chip. The cell nuclei were stained using DAPI for 5 min for cell labeling. Finally, the samples were examined using confocal microscopy. The nucleus image was excited at 408 nm, and the emission was detected from 450 nm to 500 nm. The UCNP fluorescence was excited at 808 nm. The emission was detected from 450 nm to 500 nm. To observe the NBs we added DiI inside the NB phospholipid. DiI was excited at 561 nm, and the emission was detected from 550 nm to 600 nm.

### *In vitro* cell viability and cytotoxicity of UCNP–CN@NBs with/without oxygen embedded in NBs

For the cytotoxicity assay, CAL-27 and OECM-1 cell lines were separately grown in DMEM and RPMI-1640 medium and mixed with 10% fetal bovine serum and 1% triple antibiotic reagent. Both cell lines were incubated in a 5% CO_2_ incubator at 37 °C. Briefly, each well containing 2000 cells was inoculated in a 96-well plate for 12 h. Different diluted concentrations of UCNP–CN@NBs and UCNP–CN@NBs with O_2_ (3, 9, 27, 81 and 250 μg mL^–1^) were used to treat the cells for another 24 h. Furthermore, in the NIR treatment, all cell test experiments will be exposed to a short irradiation under the 808 nm NIR laser (0.5 W cm^–2^). After 48 h incubation, measurements from the Alamar Blue assay were obtained using SpectraMax M2 (Molecular Devices, California, USA) at an excitation/emission of 560/590 nm.

### Detection of the *in vitro* photodynamic effect

Intracellular ROS generation was detected immediately after the photosensitization experiments using the reagent H_2_-DCFDA. The DMEM culture medium of the CAL-27 oral cancer cells exposed to 250 μg mL^–1^ nanocomposites (UCNP–CN@NBs) was replaced with PBS, which contained 25 μM H_2_-DCFDA and 0.1 μg mL^–1^ of DAPI and completely covered the adhering cells. The cells were then subjected to a photosensitization experiment by irradiation at 0.5 W cm^–2^ using the 808 nm laser for 10 min. The fluorescence images of the cells stained with dichlorofluorescein (DCF) (green emission at 520 nm) and DAPI (blue emission at 450 nm) were promptly captured by a Leica TCS SP5 confocal microscope after excitations at 480 and 350 nm. Cell apoptosis was detected using a terminal deoxynucleotidyl transferase deoxyuridine triphosphate nick end labeling (TUNEL) assay. The apoptosis detecting reagent, TUNEL mixture, was added to an enzyme solution to give a total volume of 50 μL, which was then added to the remaining 450 μL label solution to obtain a 500 μL TUNEL reaction mixture. The mixture was mixed well to equilibrate the components. We then detected *in situ* cell death using a confocal microscope to evaluate the grade of cell apoptosis. For evaluation by a confocal microscope, we used an excitation wavelength in the range of 450–500 nm and detected the green fluorescence in the range of 515–565 nm.

### Animal model tumor and organs imaging

An InGaAs charge-coupled device (CCD) camera was used to obtain a NIR image (Princeton Instruments) to ensure biodistribution of the 808 nm UCNP–PLL@CN conjugates *in vivo*. The 808 nm excitation light was provided by a fiber-coupled diode laser (5 W, Hi-Tech Optoelectronics Co., Ltd.) with a 4.5 cm laser extender counter. The emitted light passed through an 830 nm long-pass filter (Semrock) and was taken as the signal. Since the fluorescence imaging may be shielded by mouse fur, we use BALB/c nude mice to prevent light-shielding in this experiment. A volume of 100 μL of the UCNP–CN@NB-suspended solution (10 mg mL^–1^) was administered by intratumoral injection for the tumor imaging of the mouse tumor model.

### *In vivo* photodynamic effect

The animal work was performed at the Genomics Research Center following protocols approved by the Institutional Animal Care and Utilization Committee (IACUC) of Academia Sinica (IACUC no. 16-05-957). The mice were fed with *ad libitum* autoclaved water and an irradiated mouse diet (PicoLab, USA) and housed according to the animal care guidelines of the IACUC, Academia Sinica. CAL-27 cells (5 × 10^6^ cells/100 μL) were injected subcutaneously on the backside of the mouse. NBs and UCNP–CN@NBs (100 mg) were intratumorally injected into the tumor when the tumors had grown to 125 mm^3^. Irradiation with a 250 mW cm^–2^ 808 nm NIR-laser was performed after 12 h incubation for 30 min with 5 min intervals of cooling down and irradiation. All tumors were then acquired after the fifth week. Cross-sections of the tumors were stained using hematoxylin and eosin (H&E stain). All the stained tumor sections were scanned using a Leica Aperio AT2 scanner.

## Conclusions

In this study, we synthesize 808 nm-excited UCNPs embedded in NBs as the theranostic nanoplatform. The UCNPs and graphitic CNs were all encapsulated in the NBs. Using the combination of UCNP fluorescence imaging and ultrasonic NB diagnosis, we can achieve complementary multi-imaging. Meanwhile, CNs can absorb the upconverted energy of the UCNPs to produce ROS and cause cancer cell death. The important results of this study are as follows. (1) We combined ultrasonic diagnosis and fluorescence imaging to solve the insufficient penetration depth of fluorescence and the poor ultrasound resolution. (2) Up to 808 nm laser excitation can be a low-energy NIR light to motivate UCNPs to generate the fluorescence of ultraviolet light and visible light. The energy of the ultraviolet light can enable the inorganic photosensitizer, CNs, to produce ROS for phototherapy. (3) When the nanocomposites were embedded in the NBs, the 96.5% C_3_F_8_ and 3.5% O_2_ were enriched in the bubbles. Oxygen can be used to improve the hypoxic state of cancer cells and increase the ROS production to achieve an optimized PDT.

## Conflicts of interest

There are no conflicts to declare.

## Supplementary Material

Supplementary information
