# Supplementary material for: Nanobubble-embedded inorganic 808 nm excited upconversion nanocomposites for tumor multiple imaging and treatment
Source: Chem Sci. 2018 Feb 9;9(12):3141–51. doi: 10.1039/c8sc00108a (PMC5916109; doi:10.1039/c8sc00108a)
Supplement: Supplementary file 1 [file SC-009-C8SC00108A-s001.pdf]

Electronic Supplementary Material (ESI) for Chemical Science.
This journal is © The Royal Society of Chemistry 2018

Supporting Information for
Nanobubble-Embedded Inorganic 808 nm excited-Upconversion Nanocomposites for Tumorous Multiple Imaging and Treatment

Ming-Hsien Chan,‡[a] Yu-Ting Pan,‡[a] Yung-Chieh Chan,[b] Michael Hsiao,*[bc] Chung-Hsuan Chen,[b] Lingdong Sun,[d] and Ru-Shi Liu*[abe]

a. *Department of Chemistry, National Taiwan University, Taipei 106 Taiwan;*
b. *Genomics Research Center, Academia Sinica, Taipei 115 Taiwan;*
c. *Department of Biochemistry College of Medicine, Kaohsiung Medical University, Kaohsiung, 807 Taiwan;*
d. *Beijing National Laboratory for Molecular Sciences State Key Laboratory of Rare Earth Materials Chemistry and Applications PKU-HKU Joint Laboratory in Rare Earth Materials and Bioinorganic Chemistry College of Chemistry and Molecular Engineering, Peking University, Beijing 100871 China;*
e. *Department of Mechanical Engineering and Graduate, Institute of Manufacturing Technology, National Taipei University of Technology, Taipei, 106 Taiwan*

Corresponding authors

*E-mail: mhsiao@gate.sinica.edu.tw

*E-mail: rsliu@ntu.edu.tw

1. Synthesis and characterization of the nanobubbles.

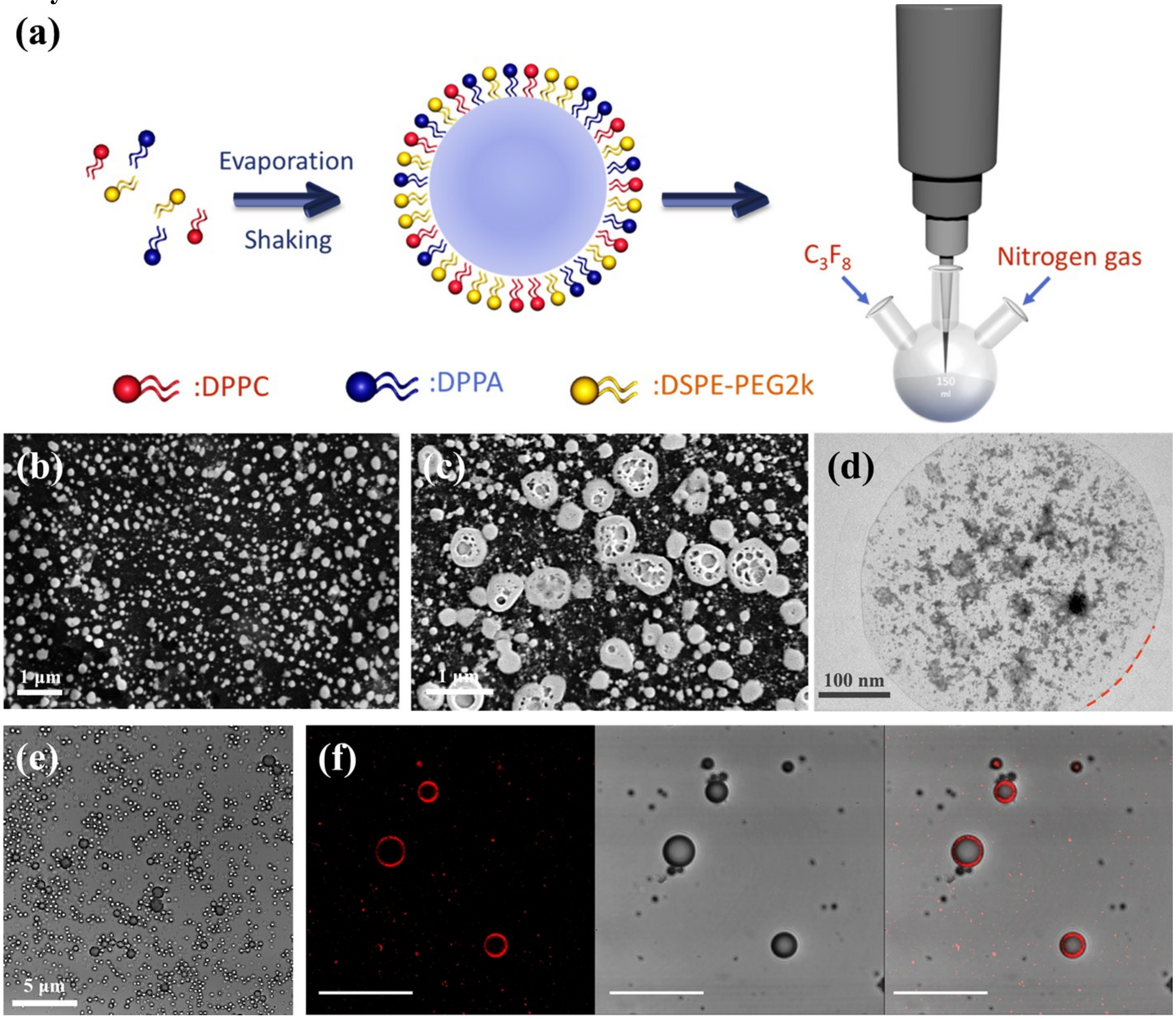

Fig. S1 (a) Synthetic process of nanobubbles (NBs) by sonication method. (b, c) Scanning electron microscopy patterns of NBs with different field scales. (d) Transmission electron microscopy imaging of NBs. (e) Confocal bright field with bubbles and (f) fluorescence imaging of bubbles loaded with DiI dye can be detected by a confocal microscope (scale bar: 1 μm).

2. Synthesis and characterization of the $NaYF_4$:Yb/Tm@$NaYF_4$:Yb/Nd upconversion nanoparticles (UCNPs) and upconversion nanoparticle-poly-l-lysine hydrobromide-carbon nitride quantum dot (UCNP–PLL@CNs) composite.

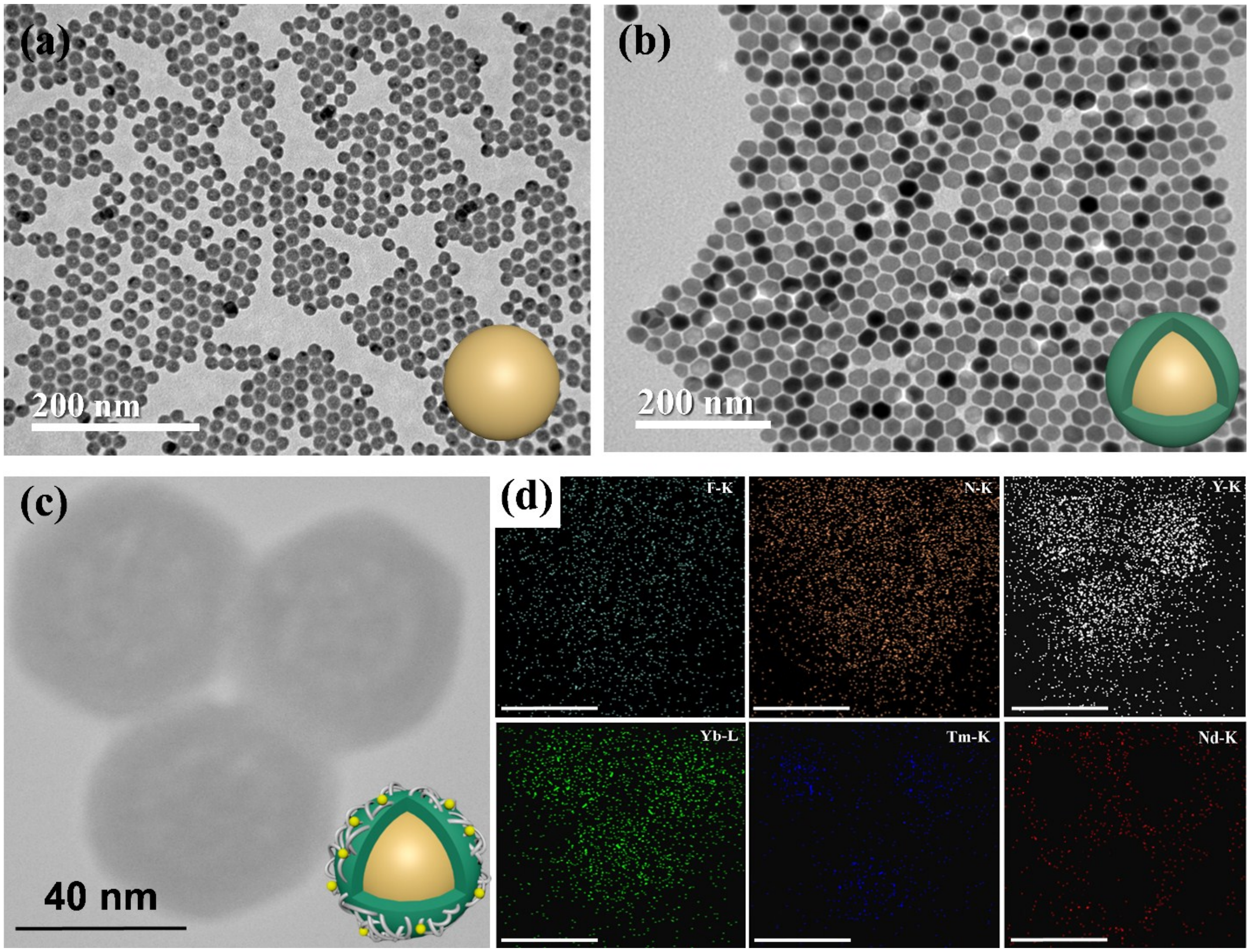

Fig. S2 TEM patterns of (a) $NaYF_4$:Yb/Tm, (b) $NaYF_4$:Yb/Tm@$NaYF_4$:Yb/Nd (UCNP), and (c) UCNP–PLL@CNs. (d) High angle annular dark field-scanning transmission electron microscopy energy-dispersive X-ray spectroscopy mapping images of UCNP–PLL@CNs to ensure the element distribution of F, Y, Yb, Tm, and Nd for UCNP and N for CNs (scale bar: 40 nm).

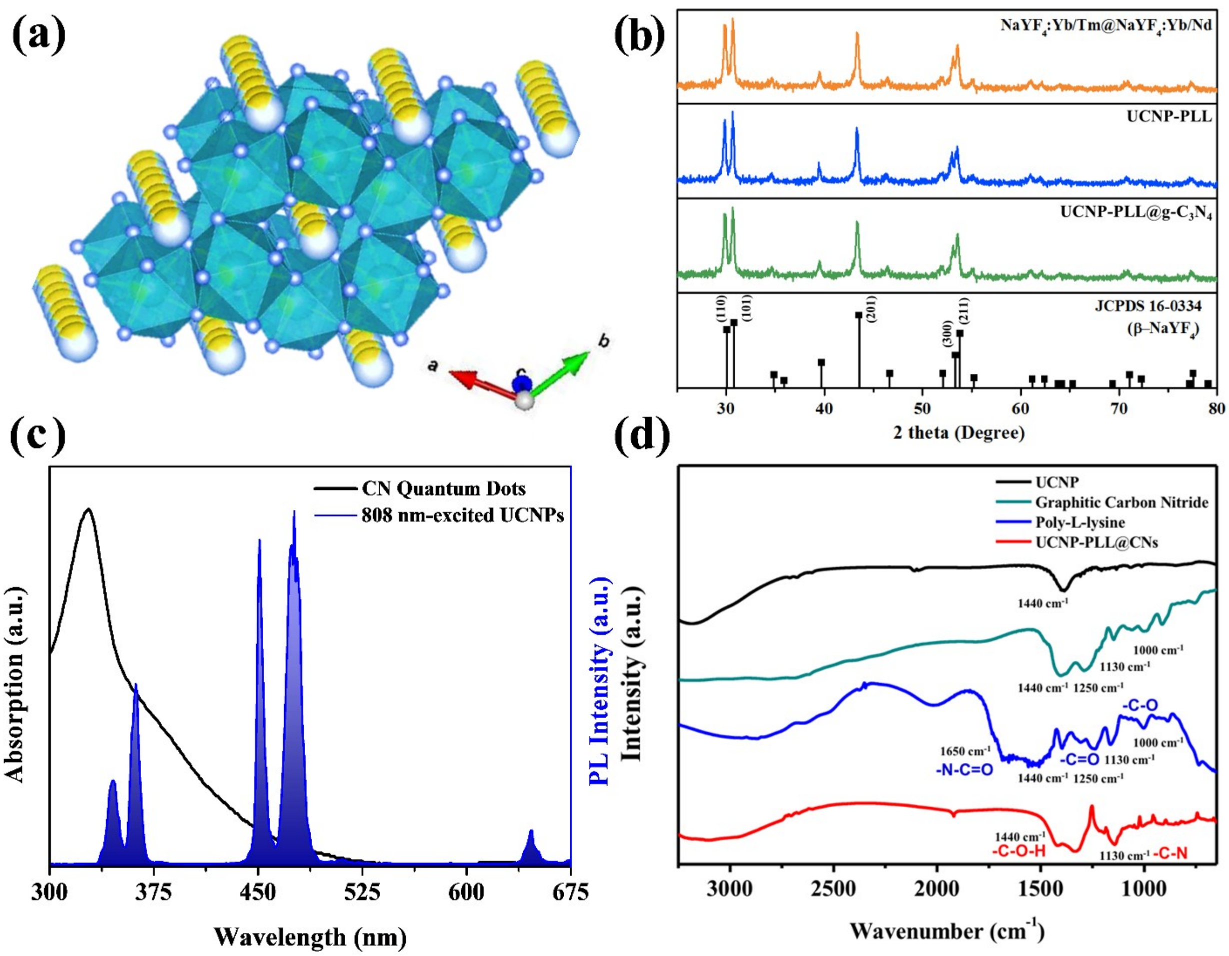

Fig. S3 (a) Crystal structure of β-$NaYF_4$ hexagonal phase and (b) X-ray diffraction patterns of $NaYF_4$:Yb/Tm@$NaYF_4$:Yb/Nd (UCNPs), UCNP–PLLs, and UCNP–PLL@CNs. (c) UV–Vis absorption of CN quantum dots and photoluminescence spectra of UCNPs. (d) Fourier transform infrared spectroscopy curves of UCNPs, CNs, PLL, and UCNP–PLL@CNs.

3. The survival time of the UCNP–CN@NBs with different time and laser treatment.

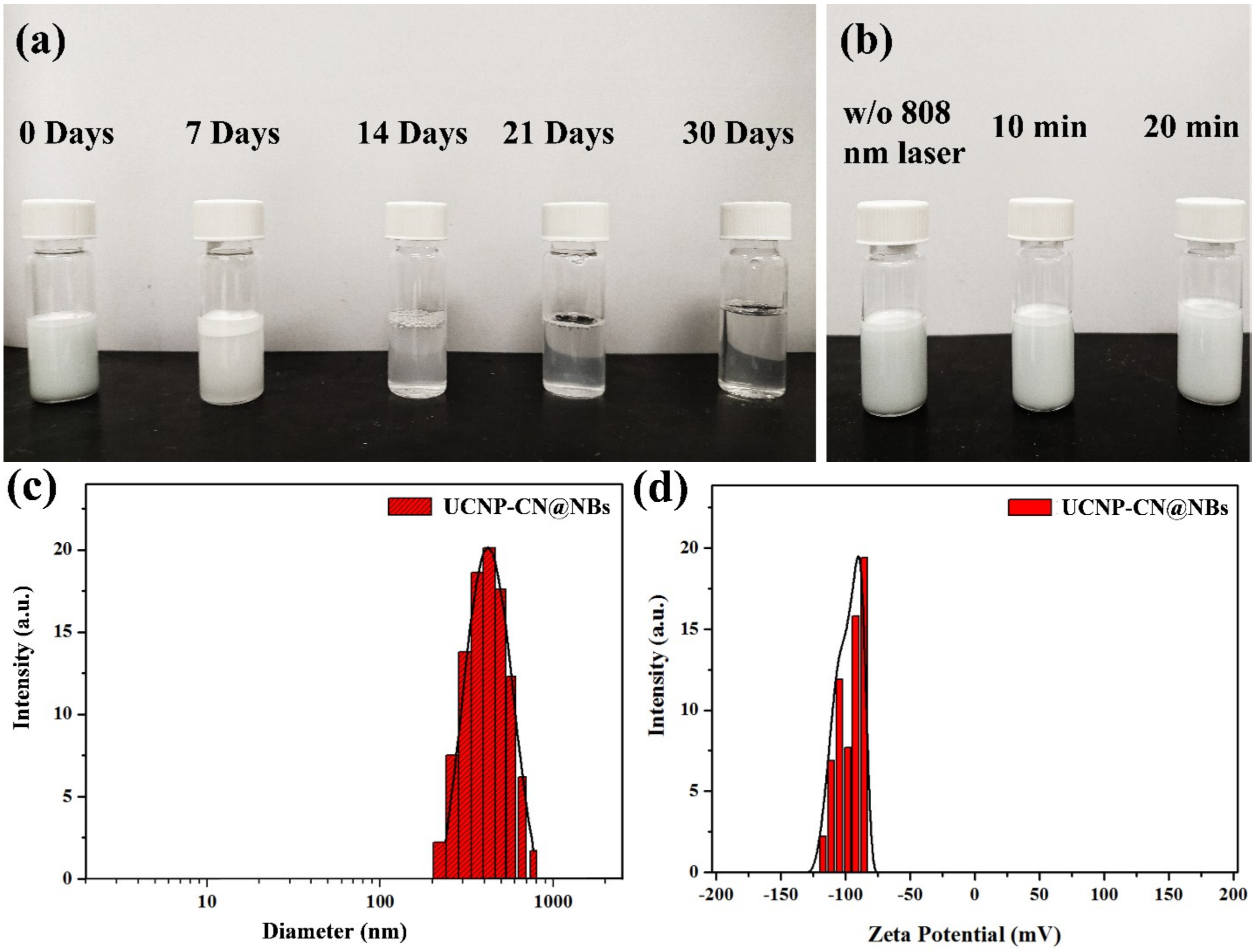

Fig. S4 UCNP–CN@NBs is reserved for (a) 0 week to 4 weeks and treated with (b) different laser usage for 0 min to 20 min. (c) Particle size analysis of UCNP–CN@NBs by dynamic light scattering. Zeta potential analysis of UCNP–CN@NBs.

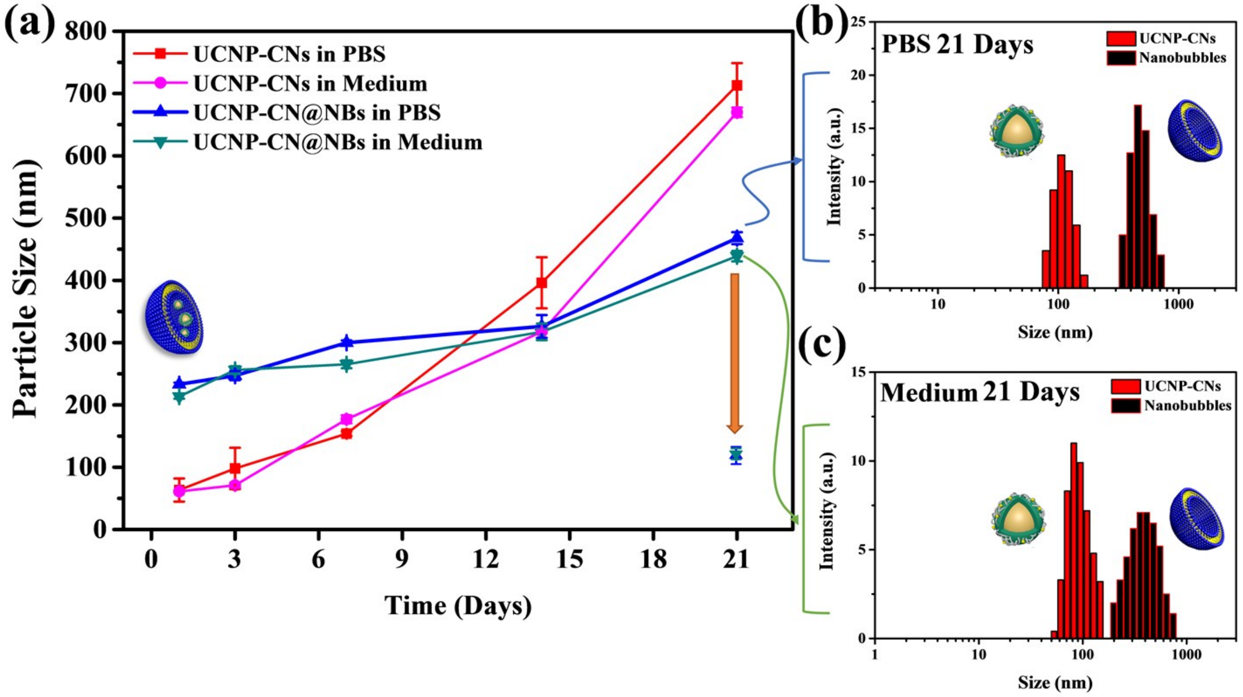

Fig. S5 UCNP–CN@NBs is reserved for (a) 0 week to 3 weeks and keep in PBS and medium solution. The size of nanobubbles and UCNP nanocomposites can be detected after keeping 21 days, respectively. The broken phenomena of UCNP–CN@NBs demonstrate that two-type distribution is present after 21 days in (b) PBS and (c) medium.

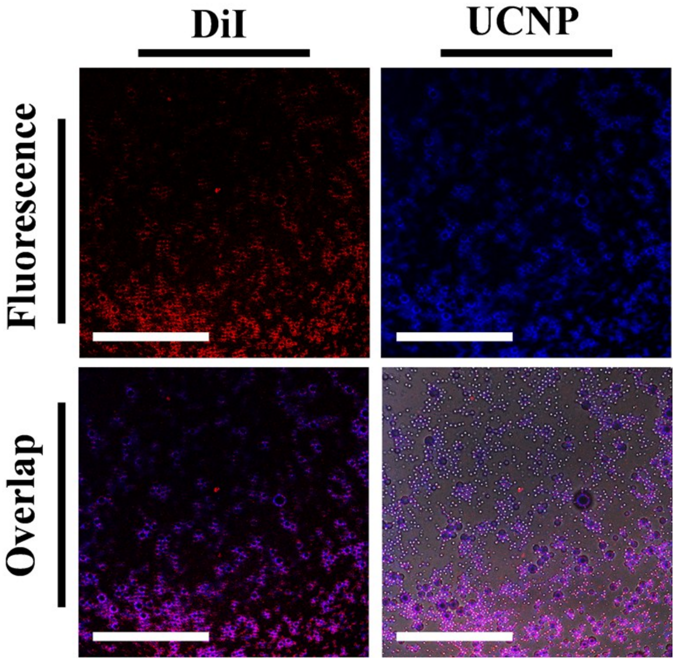

Fig. S6 The addition of the upconverted nanocomposite material cannot enter into the bubbles after forming the micelle. It can be observed from the confocal microscope diagram that the blue fluorescence is not located in the bubbles (scale bar: 10 μm).

4. *In vitro* assessment of UCNP–PLL@CNs.

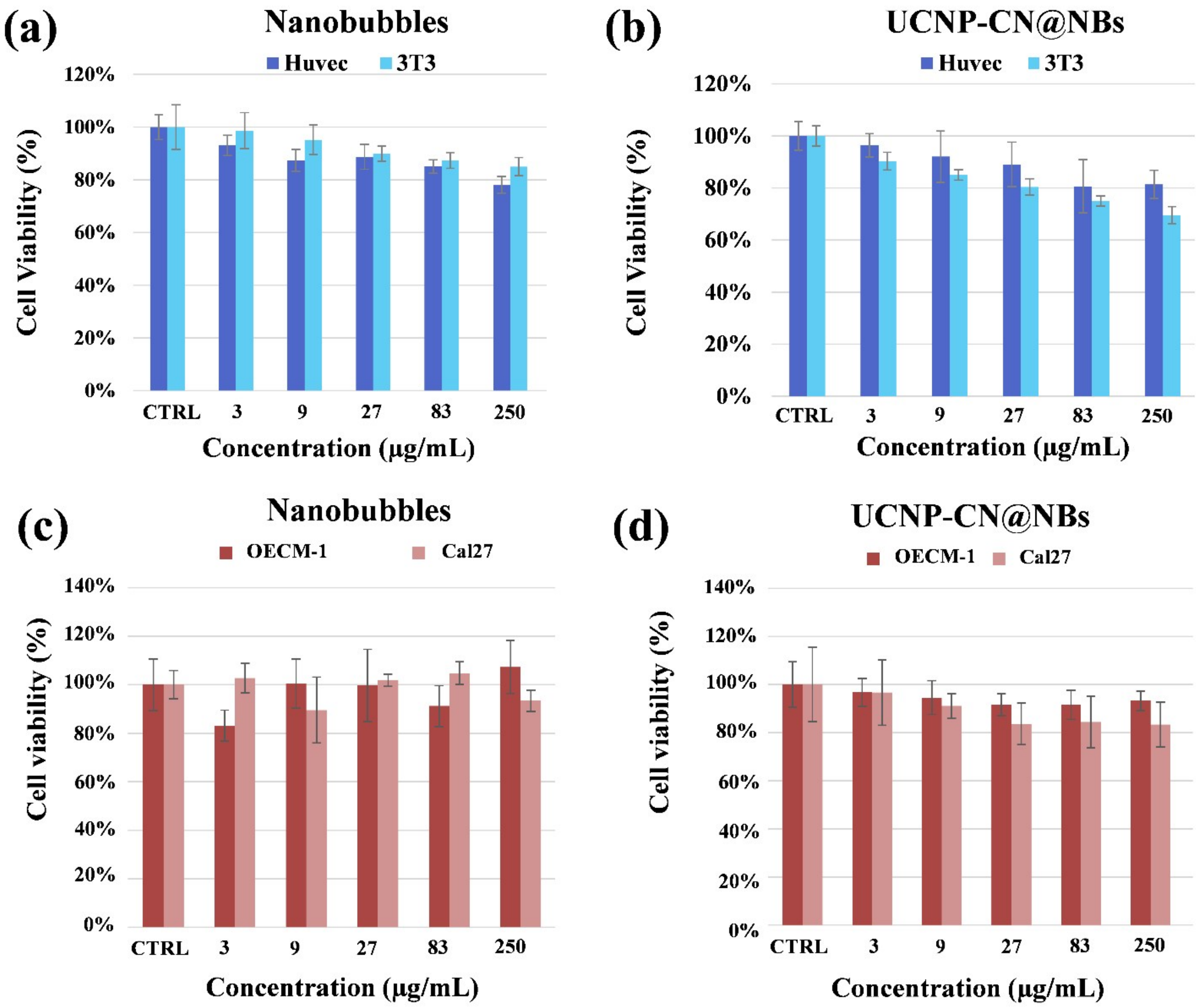

Fig. S7 Cell viability after treatment. (a) Nanobubbles (NBs) and (b) UCNP–PLL@CNs with Huvec and 3T3 normal cells. (c) NBs and (d) UCNP–PLL with OECM-1 and Cal27 cancer cells.

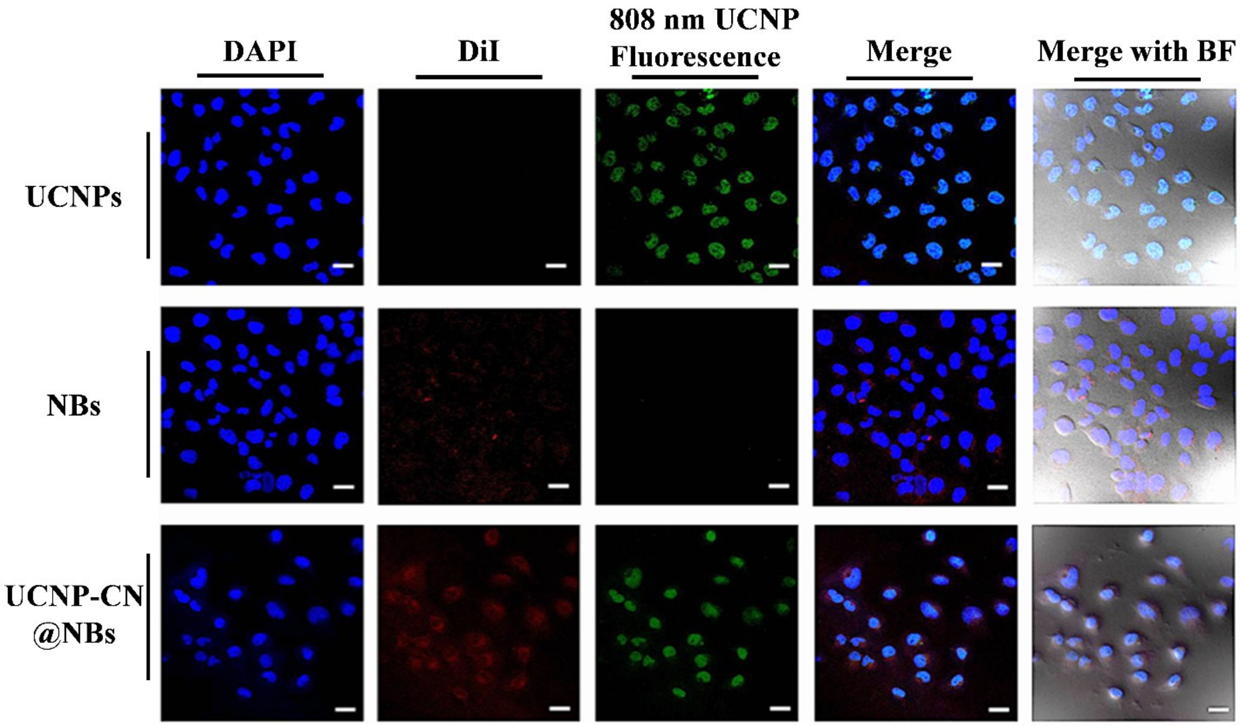

Fig. S8 Confocal images of OECM-1 treated with 808 nm UCNPs, nanobubbles (NBs), and UCNP–PLL@CNs for 12 h. 4′,6-diamidino-2-phenylindole (DAPI) was used to stain for marking the nucleus location. DiI dye was used to stain for marking the double lipid layer of NBs. The UCNP emission is 450 nm, and green fluorescence was used to avoid confusion with DAPI (scale bar: 25 μm).
